# Development of a Xeno-Free Feeder-Layer System from Human Umbilical Cord Mesenchymal Stem Cells for Prolonged Expansion of Human Induced Pluripotent Stem Cells in Culture

**DOI:** 10.1371/journal.pone.0149023

**Published:** 2016-02-16

**Authors:** Qing Zou, Mingjun Wu, Liwu Zhong, Zhaoxin Fan, Bo Zhang, Qiang Chen, Feng Ma

**Affiliations:** 1 Research Center for Stem Cell and Regenerative Medicine, Sichuan Neo-life Stem Cell Biotech INC., Chengdu, Sichuan, China; 2 Center for Stem Cell Research & Application, Institute of Blood Transfusion, Chinese Academy of Medical Sciences and Peking Union Medical College, Chengdu, Sichuan, China; 3 State Key Laboratory of Experimental Hematology, Chinese Academy of Medical Sciences and Peking Union Medical College, Tianjin, China; 4 State Key Laboratory of Biotherapy, Collaborative Innovation Center for Biotherapy, West China Hospital, Sichuan University, Chengdu, Sichuan, China; University of North Dakota, UNITED STATES

## Abstract

Various feeder layers have been extensively applied to support the prolonged growth of human pluripotent stem cells (hPSCs) for *in vitro* cultures. Among them, mouse embryonic fibroblast (MEF) and mouse fibroblast cell line (SNL) are most commonly used feeder cells for hPSCs culture. However, these feeder layers from animal usually cause immunogenic contaminations, which compromises the potential of hPSCs in clinical applications. In the present study, we tested human umbilical cord mesenchymal stem cells (hUC-MSCs) as a potent xeno-free feeder system for maintaining human induced pluripotent stem cells (hiPSCs). The hUC-MSCs showed characteristics of MSCs in xeno-free culture condition. On the mitomycin-treated hUC-MSCs feeder, hiPSCs maintained the features of undifferentiated human embryonic stem cells (hESCs), such as low efficiency of spontaneous differentiation, stable expression of stemness markers, maintenance of normal karyotypes, *in vitro* pluripotency and *in vivo* ability to form teratomas, even after a prolonged culture of more than 30 passages. Our study indicates that the xeno-free culture system may be a good candidate for growth and expansion of hiPSCs as the stepping stone for stem cell research to further develop better and safer stem cells.

## Introduction

Human pluripotent stem cells (hPSCs), including both human embryonic stem cells (hESCs) and human induced pluripotent stem cells (hiPSCs), have the unlimited self-renewal capacity and the potential to differentiate into all three germ layers-derived tissues of the human body. The hiPSCs were first directly reprogrammed from human adult somatic cells by the activation of transcription factors including OCT3/4, SOX2, c-MYC, KLF4, NANOG and LIN28 [1, 2]. Because hiPSCs skillfully overcome ethical concerns in utilization of hESCs, they provide a valuable research tool and may be an unlimited autologous cell source for research on basic biology, patient-tailored disease models, durg screening, genetic correction and cellular therapies in the future [3–7].

For the sustained maintenance, hPSCs often depend on a coculture with a feeder layer of mouse embryonic fibroblasts (MEF) or mouse fibroblast cell line (SNL), which inevitably create the risk of release animal materials as well as contamination of unknown pathogens [8, 9]. The potential risk of cross-species exposure to rodent pathogens and gene products hamper the clinical application of hPSCs. These immunogenic contaminations are difficult to eliminate from human stem cell lines cocultured on animal cells. Therefore, development of a human-source feeder is acutely needed. Various human tissue-derived feeder cells such as human foreskin fibroblasts [10–12], fetal muscle and skin fibroblast [13] and adult fallopian tube epithelial cells [13] were reported to support the growth of hESCs.

Mesenchymal stem cells (MSCs) are multipotent stromal cells and can be isolated from different tissues [14]. They possess many remarkable properties, including immunomodulation, regeneration and favoring therapeutic uses [14]. Since the first identification of human MSCs was from bone marrow (hBM-MSCs), and their properties well characterized [15], hBM-MSCs have been widely used in the past years. But the several drawbacks in collecting cells, aging, high viral pollution, requiring invasive procedure and limited proliferative property of hBM-MSCs restrict the utility in stem cells-based therapies [16, 17]. The human umbilical cord-derived MSCs (hUC-MSCs) also exhibit the characteristics of stromal cells, which have been shown to differentiate into osteocytes, adipocytes, neural-like cells and hepatocyte-like cells in vitro [18–20], possessing immunosuppression and hematopoiesis-supportive function [21, 22]. Furthermore, the hUC-MSCs can be obtained from umbilical cord through noninvasive procedures. Previous study has demonstrated that hESCs had been continuously cocultured with hUC-MSCs feeder in vitro, but these cells gradually lost the potential for teratoma formation [23].

Recently, we have keen focusing our attention on research of human (and non-human primates) PSCs and their hematopoietic derivations [24–28]. We are trying to establish a xeno-free feeder layer system from hUC-MSCs for the prolonged expansion of hiPSCs in culture. In this study, we found although about sixty percent of hiPSC colonies spontaneously differentiated after passage onto the hUC-MSCs feeder from MEF feeder, the hiPSCs adapted to the new xeno-free feeder as they gradually decreased the number of differentiated colonies. Importantly, hiPSCs maintained the features of undifferentiated hESCs on the mitomycin-treated hUC-MSCs feeder after a prolonged culture of more than 30 passages. To our knowledge, this is the first report to successfully use hUC-MSCs for the prolonged culture of hiPSCs.

## Materials and Methods

### Isolation and culture of hUC-MSCs

Human umbilical cords (hUCs) were collected from West China Women’s and Children’s Hospital without any complications of pregnancy or parturition, and the collected hUCs were transferred in sterile boxes that contained cold Hanks’ Balanced Salt Solution (HBSS) (Life Technologies, USA). Written informed consent was obtained from the pregnant women before labor. This study was approved by the institutional ethics committee of Institute of Blood Transfusion, Chinese Academy of Medical Sciences and Peking Union Medical College (CAMS & PUMC).

hUCs were dissected longitudinally, and the arteries and veins were removed. The remaining pieces were chopped into 0.2cm^3^ size. These explants were transferred in the CELLstart™ CTS™ Substrate (Life Technologies, USA)-coated 100 mm plates (Nunc, Denmark). Then, a few StemPro® MSC SFM XenoFree medium (Life Technologies, USA) with 1% penicillin-streptomycin (Life Technologies, USA) was added to the plates, and the explants were cultured at 37°C in a 5% CO_2_ incubator and left undisturbed to allow the cells to migrate from the explants. After 7–9 days, the MSC-like cells were found around the fragments. The cells were passaged into another plates and further split 1:4 by 0.05% Trypsin-EDTA (Life Technologies, USA) once the cells reached 80% confluence.

### Freezing and thawing protocols of hUC-MSCs

Once hUC-MSCs reached 80% confluence, these cells were treated with 0.05% Trypsin-EDTA and centrifuged at 200 g for 5 minutes. Then, the cell pellet were resuspended in ice-cold freezing medium including 90% medium and 10% DMSO (Life Technologies, USA). cells were quickly placed in cryovials (Nunc, Denmark) within cryofreezing container. Once frozen, the cells were transferred to -80°C overnight and retransferred to liquid nitrogen tank vapor phase for long-term storage.

When the cells were prepared for thawing, the cryovial was immersed in a 37°C water bath and swirled gently, then cells were gently pipeted into a conical tube containing medium and centrifuged at 200 g for 5 minutes. The cell pellet were resuspended in medium and transferred to CELLstart™ CTS™ Substrate -coated dishes, and incubated at 37°C in a 5% CO_2_ incubator.

### Flow cytometry analysis

Flow cytometry analysis was performed in order to characterize the hUC-MSCs. A total of 5 × 10^5^ cells were divided into aliquots in amber-tinted 1.5 ml microcentrifuge tubes (Axygen, USA). To block non-specific binding, a solution of 3% human serum in Phosphate-Buffered Saline (PBS) was added to cell suspension for 30 minutes. Then cells were resuspended in 1 ml PBS and pelleted by centrifugation for 5 minutes at 300 g. The following cell surface epitopes were detected with anti-human CD73-PE, CD90-FITC, CD105-PE, CD34-FITC, CD45-PE and HLA-DR-FITC (BD Biosciences, USA). Appropriate isotype controls were used for each antibody to assess nonspecific antibody binding. Then cells were analyzed using flow cytometry instrument (FC500; Beckman Coulter, USA) and data processing software (FlowJo 10.0.7; TreeStar, USA).

### *In vitro* differentiation assay for hUC-MSCs

To induce osteogenic and adipogenic differentiations in vitro, the hUC-MSCs were cultured using by the osteogenic medium (MSC medium supplemented with 0.1 μM dexamethasone, 10 mM β-glycerol phosphate, 50 μM ascorbate and 1% penicillin-streptomycin) and the adipogenic medium (MSC medium supplemented with 1 μM dexamethasone, 5 μg/mL insulin, 0.5 mM isobutylmethylxanthine, 60 mM indomethacin and 1% penicillin-streptomycin) respectively. After 20 days, the potential for osteogenesis was evaluated by determining the mineralization of calcium by staining with Alizarin Red S (Sigma-Aldrich, USA). The potential for adipogenesis was detected by determining the intracellular lipid droplets by staining with Oil Red O (Sigma-Aldrich, USA).

### Preparation of hUC-MSCs for the xeno-free feeder

Cultured hUC-MSCs (3–7 passages) were mitotically inactivated with 10 μg/mL mitomycin C (MMC) (Sigma-Aldrich, USA) for 3 hours at 37°C and washed three times with PBS. MMC-treated hUC-MSCs were then planted on CELLstart™ CTS™ Substrate -coated 100mm culture plates (Nunc, Denmark) at a density of 2.7 × 10^4^ cells per square centimetre.

### Induction and culture of hiPSCs

The hiPSC lines were derived from human dermal fibroblasts (HDF) using exogenous factors (OCT4, SOX2, c-MYC and KLF4) (Sidansai, China). Briefly, 10,000 cells per plate from HDF at early passage were infected with pVSVG-based lentiviruses (Sidansai, China) at a multiplicity of infection of 5 in 100 mm culture plates (Nunc, Denmark). After infection, the virus-containing solution was removed, and cells were cultured in hiPSCs medium containing 1:1 Dulbecco's modified Eagle's medium/F-12 (Life Technologies, USA), 20% knockout-replacement serum (Life Technologies, USA), 1× nonessential amino acids (Life Technologies, USA), 2 mM glutamine (Life Technologies, USA), 0.1 mM β-mercaptoethanol (Sigma-Aldrich, USA), 10 ng/mL basic fibroblast growth factor (bFGF) (Peprotech, USA) and 1% penicillin-streptomycin (Life Technologies, USA). The infected cells were transferred to 100 mm plates filled with MMC-treated MEF and cultured at 37°C in 5% CO_2_ with a medium changed daily. Morphologically undifferentiated, colony-forming cells were picked and dissociated mechanically into small clumps with a micropipette tip 3–4 weeks after infection. When the hiPSC lines were established, either MEF and hUC-MSCs after MMC deactivation were used as feeder cells for continuously subculturing of hiPSCs. The hiPSC lines were freezed and thawed according to Holm’s method [29].

### Karyotype analysis

To analyze the karyotype of the hiPSCs continuously subcultured for more than 30 passages on the hUC-MSCs feeder, cell division was blocked at metaphase with 0.1 μg/mL colcemid (Calbiochem, Germany) for 2 hours at 37°C. The cells were washed and trypsinized, resuspended in 0.075 M KCl, incubated for 20 minutes at 37°C, and fixed with methanol and acetic acid (3:1). G-banding standard staining was used to visualize the chromosomes. At least 20 metaphase-nuclei were detected. The metaphases were analyzed and reported by a certified cytogenetic laboratory according to the International System for Human Cytogenetic Nomenclature.

### RNA preparation, reverse transcription and quantitative Real Time-Polymerase Chain Reaction (qRT-PCR)

Total RNA was isolated from undifferentiated hiPSCs on different feeders with Trizol reagent (Life Technologies, USA) respectively. The concentration of isolated total RNA was determined by UV-Vis Spectrophotometer (NanoDrop 2000; Thermo Fisher Scientific, USA). The first strand of complementary DNA (cDNA) was synthesized using Transcriptor cDNA Synth Kit (Roche, USA) with oligodT primers following the manufacturer’s instructions. Primer sequences are shown in Table 1.

**Table 1:** Primer sequences.

Primer	Sequence(5' to 3')	Product Size (bp)
Exogenous-OCT4-F	ACTGTACTCCTCGGTCCCT	167
Exogenous-OCT4-R	ACACCGGCCTTATTCCAAGC	
Exogenous-SOX2-F	CACATGTCCCAGCACTACCAG	169
Exogenous-SOX2-R	ATAGACAAACGCACACCGGCCTT	
Exogenous-C-MYC-F	ATACATCCTGTCCGTCCAAGC	197
Exogenous-C-MYC-R	ACACCGGCCTTATTCCAAGC	
Exogenous-KIF4-F	AACTGACCAGGCACTACCGTA	196
Exogenous-KIF4-R	CACACCGGCCTTATTCCAAGC	
OCT4-F	CTTGCTGCAGAAGTGGGTGGAGGAA	169
OCT4-R	CTGCAGTGTGGGTTTCGGGCA	
SOX2-F	CAGCCCATGCACCGCTACGAC	163
SOX2-R	TGGCCTCGGACTTGACCACC	
MYC-F	CCCTGCGTGACCAGATCCC	160
MYC-R	TGTTTCAACTGTTCTCGTCGTT	
NANOG-F	CAAAGGCAAACAACCCACT	76
NANOG-R	CTTCTGTTTCTTGACCGGGAC	
REX1-F	CGCCCACAGTCCATCCTTACAGA	183
REX1-R	TCATAGGCCACCTTCTCCTCGTT	
GAPDH-F	AACGGATTTGGTCGTATTG	135
GAPDH-R	ATGGGTGGAATCATATTGG	
BS-OCT4-F	GAGGTTGGAGTAGAAGGATTGTT	461
BS-OCT4-R	TAACCCATCACCTCCACCAC	
BS-NANOG-F	GGTTAGGTTGGTTTTAAATTTTTG	341
BS-NANOG-R	CCTACTAACCCACCCTTATAAATTC	

OCT4: octamer-binding transcription factor 4; SOX2: SRY related HMG box-2; REX1: reduced expression protein-1; GAPDH: glyceraldehyde-3-phosphate dehydrogenase; BS: bisulfite sequencing.

For qRT-PCR analysis, these cDNA were used as the templates with FastStart universal SYBR green master (ROX) (Roche, USA) in the Real-time PCR Detection System (IQ5; Bio-rad, USA). The gene of GAPDH (glyceraldehyde-3-phosphate dehydrogenase) was used as an endogenous reference. The No Template Control (NTC) contained the sterilized water instead of cDNA template. The conditions for PCR reactions were as following: denaturation at 95°C for 2 minutes, 45 cycles of denaturation at 95°C for 30 seconds, annealing at 56°C for 30 seconds and extension at 72°C for 30 seconds. The relative expression levels of genes were determined according to the delta-delta Ct method. The results were analyzed using GraphPad Prism 6 (GraphPad Software, USA) and SPSS Statistics 20.0 (IBM, USA).

### Characterization of hiPSCs

The hiPSCs were continuously subcultured for more than 30 passages on hUC-MSCs feeder and characterized by immunocytochemistry with stemness-related fluorescence-labeled antibodies, which were OCT4 (octamer-binding transcription factor 4), SOX2 (SRY related HMG box-2), NANOG, SSEA4 (stage-specific embry-onic antigen-4) and TRA-1-60 (Abcam, UK). The nuclei were visualized by staining with DAPI (4’,6-Diamidine-2’-phenylindole dihydrochloride) (Roche, USA).

The hiPSCs were cultured on sterile cover glass in 24-well plates (Nunc, Denmark) with the hUC-MSCs feeder. After 2–3 days of following passage, growing hiPSCs on the feeder were washed with PBS and fixed in 4% paraformaldehyde (Boster, China), then the cells were incubated with the following antibodies: OCT4, SOX2, NANOG, SSEA4 and TRA-1-60 (Abcam, UK), and stained according to manufacturer’s protocol. The stained cells were imaged with an inverted fluorescence microscope (ix71; Olympus, Japan).

### Bisulphite genomic sequencing

Bisulphite treatment of genomic DNA (gDNA) was carried out with an EpiTect Fast Bisulfite Conversion Kit (QIAGEN, Germany) according to the manufacturer’s protocol. Sample treatment and processing were performed simultaneously for hiPSCs and parental cells. The promoter regions of the human OCT4 and NANOG genes were amplified by PCR. The PCR products were subcloned into pGM-T. Ten clones of each sample were verified by sequencing with the M13 universal primer. Primer sequences used for PCR amplification were provided in Table 1.

### *In vitro* differentiation potentials

For Embryoid Bodies (EBs) formation, hiPSCs cocultured on hUC-MSCs feeder were harvested by treating with dispase (Life Technologies, USA). The clumps of the cells were transferred to low-attachment six-well plates (Corning, USA) in Iscove's Modified Dulbecco's Medium (IMDM) (Life Technologies, USA) containing 10% FBS (Life Technologies, USA), 100 ng/ml recombinant human stem cell factor (SCF) (Peprotech, USA), 100 ng/ml fms-related tyrosine kinase 3 ligand (FLT-3L) (Peprotech, USA), 20 ng/ml bone morphogenetic protein 4 (BMP4) (Peprotech, USA) and 1% penicillin-streptomycin.

The hiPSCs were cultured on the hUC-MSCs feeder for 6–7 days after 30 passages. Then, the hiPSC colonies were picked up mechanically and dissociated with Accutase solution (Life Technologies, USA), and these cells were plated onto the Cultrex PathClear BME (R&D Systems, USA)-coated 24-well plates (Nunc, Denmark) at a density of 1.1 × 10^5^ cells per square centimetre. While the hiPSCs reached approximately 50% confluence in each well, these cells were cultured with endoderm differentiation medium, ectoderm differentiation medium and mesoderm differentiation medium (Human Pluripotent Stem Cell Functional Identification Kit; R&D Systems, USA) respectively instead of hiPSCs medium according to manufacturer’s protocol for 2–3 days. To assess the expression of differentiation markers specific for each of the three germ layers, the cells were incubated with goat anti-human SOX17, goat anti-human Otx2 and goat anti-human Brachyury (Abcam, UK), respectively. The nuclei were visualized by staining with DAPI.

### Teratoma formation

To analyze the in vivo pluripotency of hiPSCs cultured on hUC-MSCs feeder, the hiPSCs were harvested by collagenase IV (Stemcell, Canada) treatment and were injected subcutaneously to the dorsal flank of 4-week-old nonobese diabetic/severe combined immunodeficiency (NOD/SCID) mice according to Yamanaka’s method[1]. Twelve weeks after the injection, the NOD/SCID mice were sacrificed and the masses were sectioned. The masses were fixed in 4% paraformaldehyde and stained with hematoxylin and eosin (HE) (Sigma-Aldrich, USA). The stained tissues were examined histologically.

## Results

### Characterization of isolated hUC-MSCs

**Fig 1:**
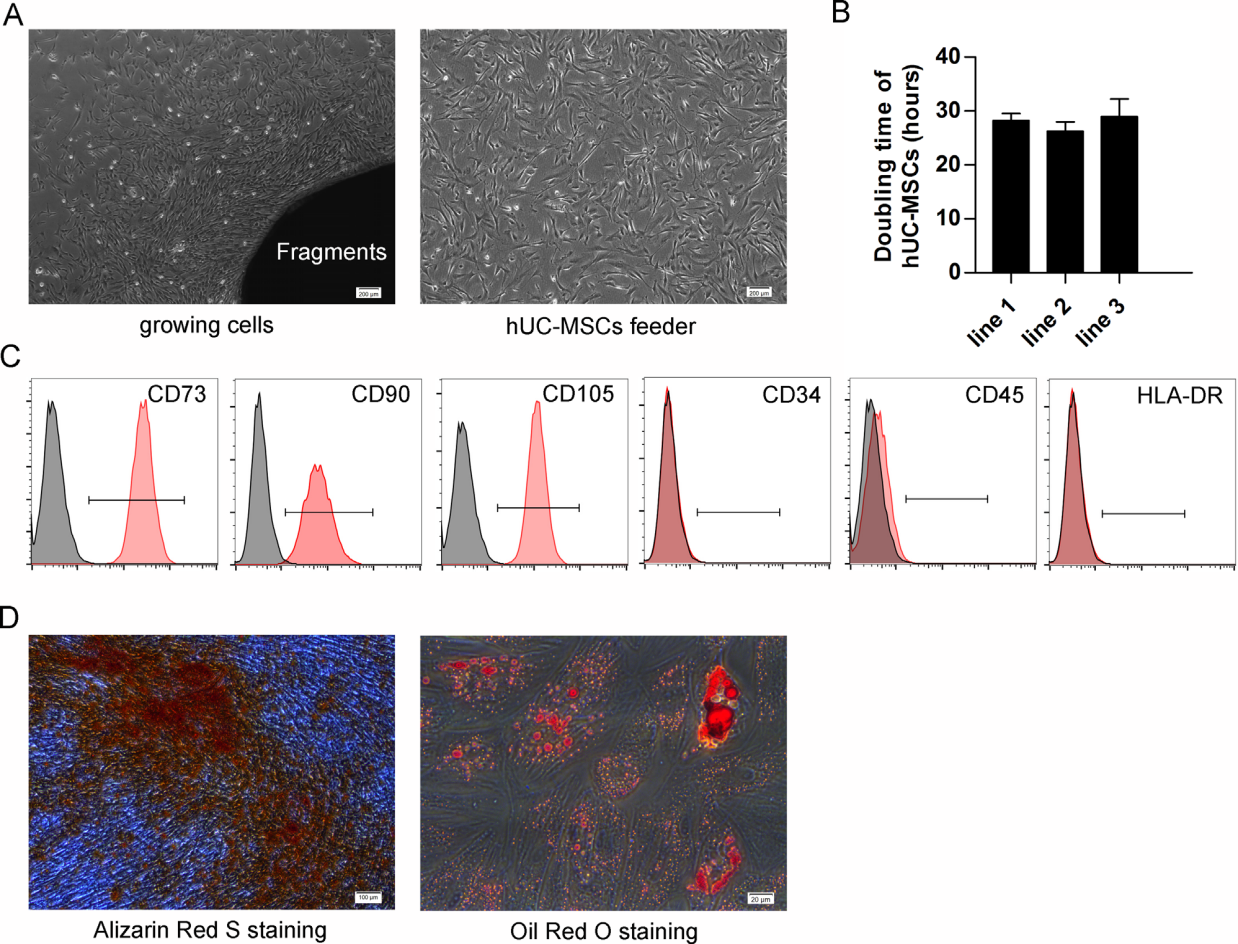
Characterization of hUC-MSCs. (A) Growing cells from fragments (left) and MMC-treated hUC-MSCs feeder (right) show property of fibroblast-like cells with a spindle-shaped morphology. Scale bar: 200 μm. (B) The doubling time of different hUC-MSC lines. Each column represented the mean ±SD. (C) Flow cytometry results of rapidly dividing hUC-MSCs show they are positive for MSCs-specific markers (CD73, CD90 and CD105) but negative for CD34, CD45 and HLA-DR. (D) Alizarin Red S staining of osteogenic cells differentiated from hUC-MSCs (left). Scale bar: 100 μm. Oil Red O staining of adipogenic cells differentiated from hUC-MSCs (right). Scale bar: 20 μm.

Isolated hUC-MSCs and feeder displayed the fibroblastic morphology and were spindle-shaped (Fig 1A). The calculated population doubling time of hUC-MSC lines were 28.2 ± 1.3 (line 1), 26.3 ± 1.7 (line 2) and 28.9 ± 3.3 (line 3) hours, P>0.05 respectively (Fig 1B). Flow cytometric analysis revealed that the hUC-MSCs expressed a set of cluster of differentiation (CD) MSC markers (CD73, CD90, and CD105), furthermore, they did not express hematopoietic markers (CD34 and CD45) or human leukocyte antigen (HLA-DR) (Fig 1C).

### *In vitro* differentiation of hUC-MSCs

For osteogenic differentiation, the cells were cultured for 20 days in osteogenic medium. Calcium precipitation, determined by Alizarin Red S staining, was observed in the hUC-MSCs (Fig 1D). Adipogenic differentiation of hUC-MSCs was apparent 14 days after incubation with an adipogenic medium. The cells contained many Oil Red O-positive lipid droplets (Fig 1D).

### Prolonged expansion of hiPSCs cocultured on hUC-MSCs

**Fig 2:**
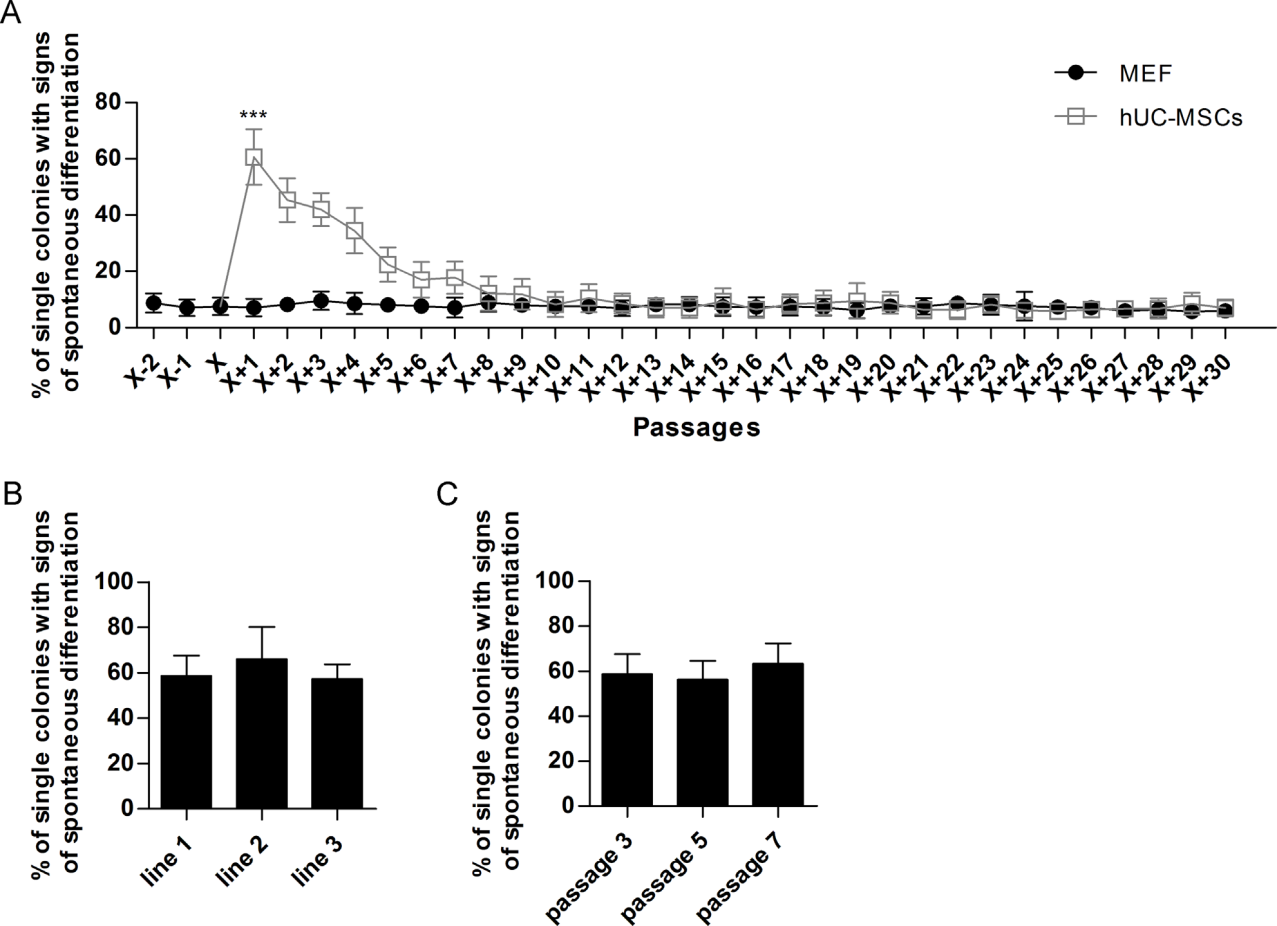
Quantification of spontaneous differentiation. (A) hiPSC colonies were derived from coculture with MEF feeder and converted to hUC-MSCs feeder at passage X (roman numeral X, is equivalent to arabic numeral 10). At each data point, a total of 100 hiPSC colonies were counted for their percentages of spontaneous differentiation. Each point represented the mean ±SD. ***, P<0.001. (B) The percentages of spontaneously differentiated hiPSCs cocultured on different hUC-MSC lines at passage X+1. Each column represented the mean ±SD. (C) The percentages of spontaneously differentiated hiPSCs cocultured on different passages hUC-MSCs feeder at passage X+1. Each column represented the mean ±SD.

**Fig 3:**
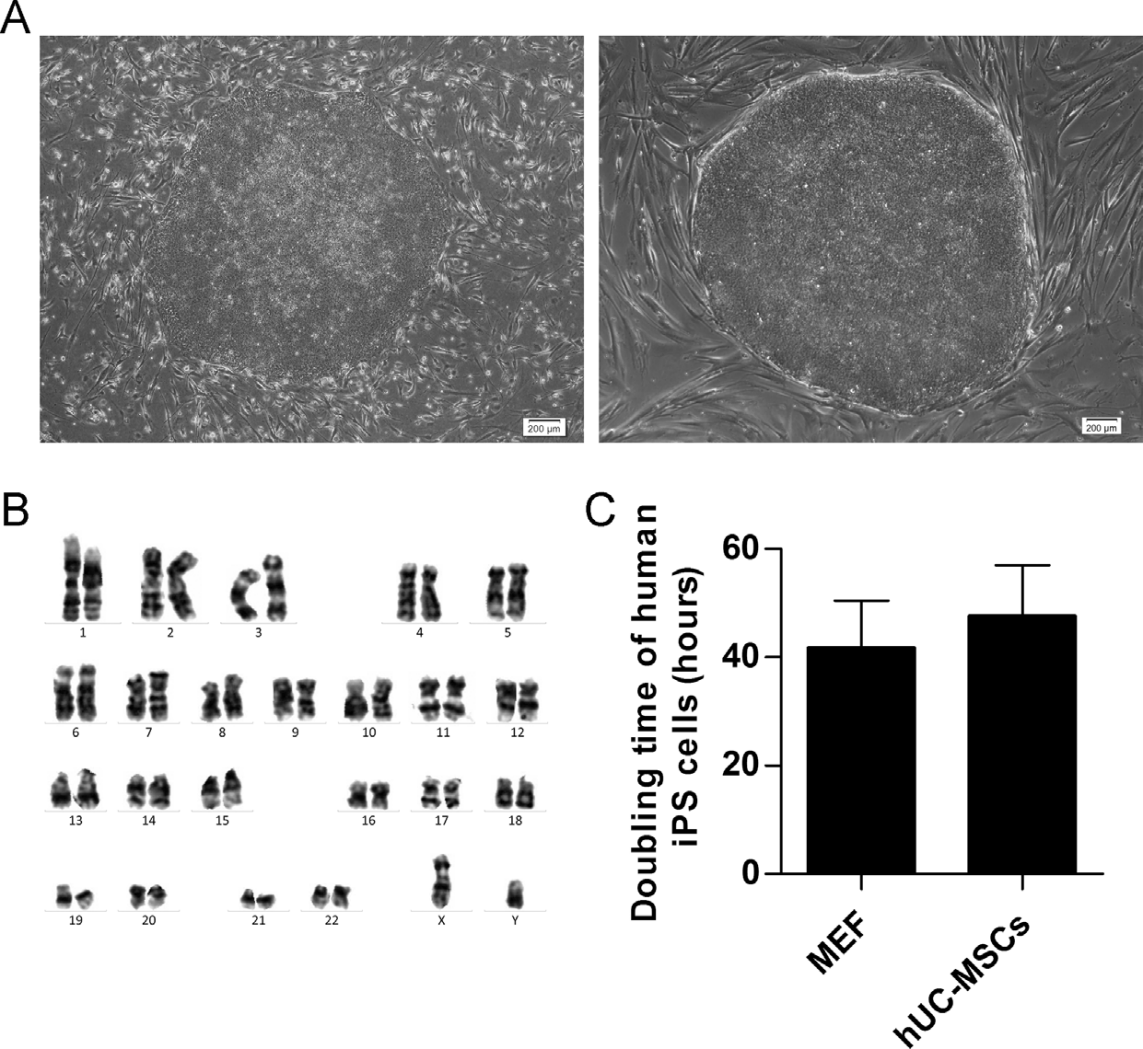
Morphology, Karyotype analysis and doubling time of human iPS cells. (A) hiPSC colonies grown on MEF (left) and hUC-MSC (right) feeders at passage X+31 revealed undifferentiated hESC morphology with high nucleus/cytoplasm ratio. Scale bar: 200 μm. (B) G-band staining showed that hiPSC cells on hUC-MSCs feeder layers at passage X+31 maintained a normal karyotype. (C) The doubling time of hiPSCs cocultured on different feeders at passage X+31. Each column represented the mean ±SD.

The undifferentiated hiPSC colonies were derived from the coculture on MEF feeder and switched to grow on the hUC-MSCs feeder layer at passage X (roman numeral X, is equivalent to arabic numeral 10). Results indicated that hiPSCs can be incessantly cultured on hUC-MSCs feeder. About sixty percent of hiPSC colonies spontaneously differentiated after being passaged onto the hUC-MSCs feeder from the MEF feeder at passage X+1, which was significantly higher than cells that were cultured on MEF feeder (P<0.001), but the cells gradually adapted to the new system of xeno-free feeder layer because the number of differentiated colonies gradually decreased (Fig 2A). The hiPSCs fully adapted to the new feeder condition after eighth passage since at that time point, they showed similar percentages of spontaneously differentiated colonies compared to traditional MEF feeder system (Fig 2A). The percentages of spontaneously differentiated hiPSCs cocultured on different hUC-MSC lines after being passaged onto the hUC-MSCs feeder from the MEF feeder at passage X+1 were 58.7 ± 9.0% (line 1), 66.0 ± 14.2% (line 2) and 57.3 ± 6.5% (line 3), P>0.05, respectively (Fig 2B), and the percentages of spontaneously differentiated hiPSCs cocultured on different passages hUC-MSCs feeder after being passaged onto the hUC-MSCs feeder from the MEF feeder at passage X+1 were 58.7 ± 9.0% (passage 3), 56.3 ± 8.3% (passage 5) and 63.3 ± 9.1% (passage 7), P>0.05, respectively (Fig 2C). It indicated that different lines and different passages of hUC-MSCs possess similar effect in supporting hiPSCs. Furthermore, the hiPSCs can maintain undifferentiated morphology beyond 30 passages both on MEF feeder and on hUC-MSCs feeder, respectively (Fig 3A).

### Characterization of hiPSCs cocultured on hUC-MSCs

After 30 passages, the undifferentiated hiPSCs showed typical hESCs morphology (e.g. compact colonies, high nucleus-to-cytoplasm ratios and prominent nucleoli) on hUC-MSCs feeder (Fig 3A), and had normal karyotypes (46, XY) (Fig 3B).

The doubling time of human iPS cells was performed to analyze the growth efficiency of undifferentiated iPS cells on different feeders. The calculated population doubling time of human iPS cells were 41.8 ± 8.6 (cocultured on MEF feeder) and 47.7 ± 9.3 (cocultured on hUC-MSCs feeder) hours, P>0.05, respectively (Fig 3C). These results are equivalent to the reported doubling time of hiPSC cells [1].

**Fig 4:**
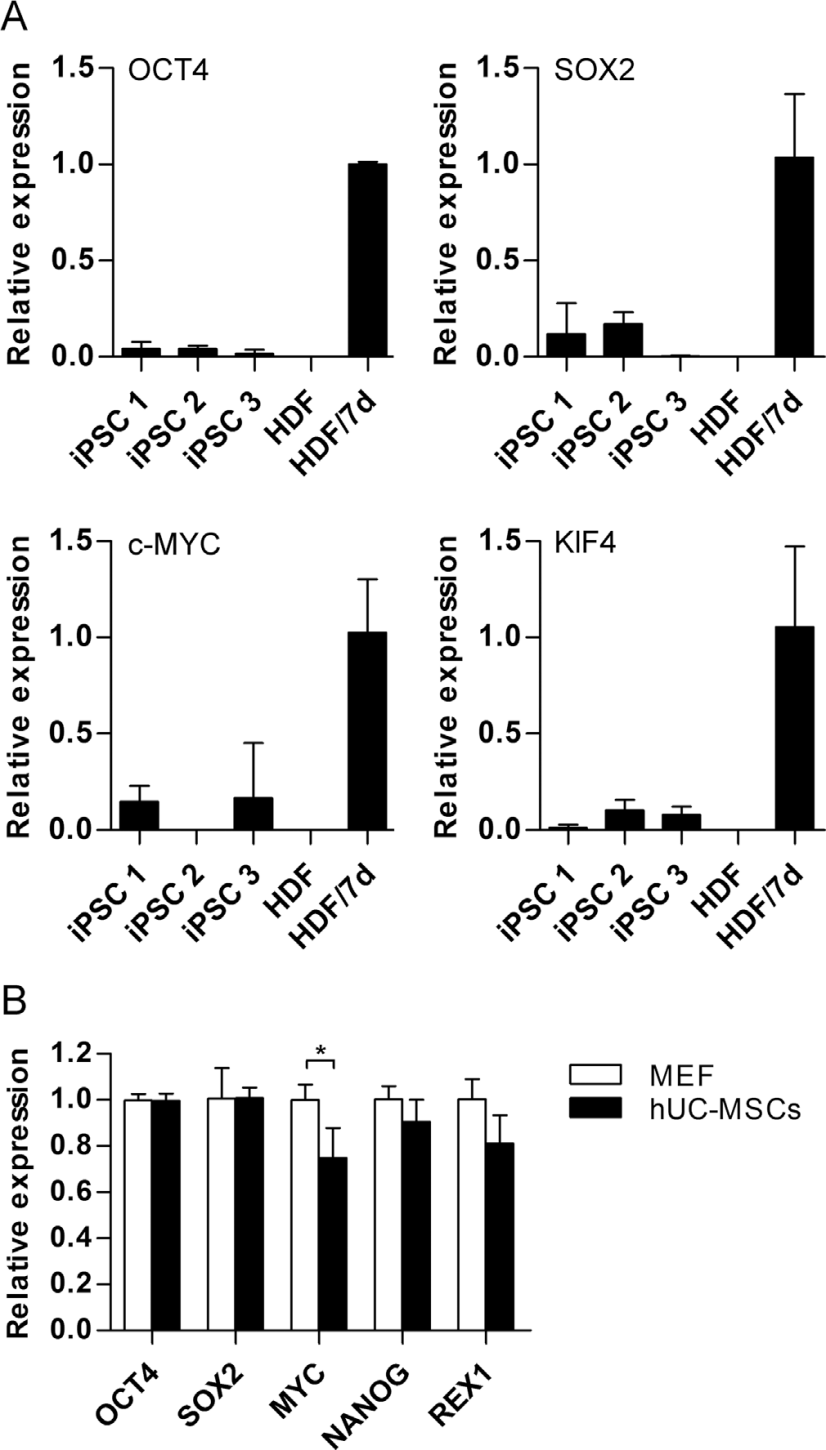
Gene expression in hiPSCs. (A) Quantitative PCR for expression of lentiviral transgenes in different hiPSC lines, HDF and HDF 7 days after the transduction with the lentiviruses. GAPDH was used as an endogenous reference. Each column represented the mean ±SD. (B) Quantitative transcriptional analysis of some pluripotent markers in hiPSCs cocultured on hUC-MSCs or MEF feeders respectively. GAPDH was used as an endogenous reference. Each column represented the mean ±SD. *, P<0.05.

The silence of the reprogramming factors was carried out to measure the reprogramming system. Quantification RT-PCR using primers specific for lentiviral transcripts confirmed efficient silencing of the lentiviruses (Fig 4A).

The expression of key pluripotency genes in hiPSCs on the two different feeder layers was further detected. The expression levels of the markers of undifferentiated stem cells, such as OCT4, SOX2, MYC, NANOG and REX1 were measured by qRT-PCR (Fig 4B). Four key pluripotency genes (OCT4, SOX2, NANOG and REX1) showed similar expression levels on the two different feeders. However, lower expression of MYC (P<0.05) was observed in hiPSCs cocultured with hUC-MSCs.

**Fig 5:**
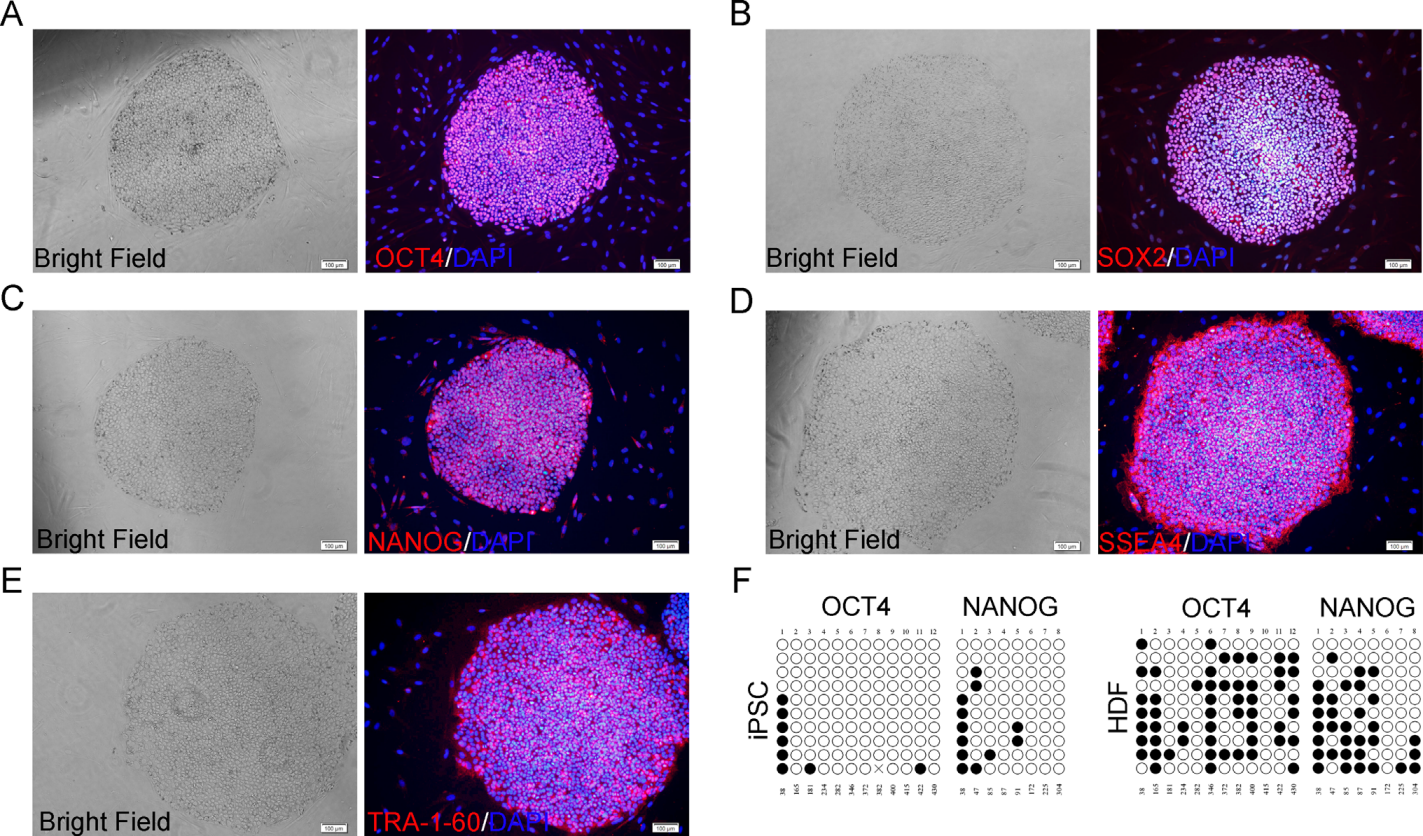
Expression of pluripotent markers and bisulphite sequencing analysis in hiPSCs on hUC-MSCs feeder. Immunocytochemistry staining showed expression of a panel of pluripotent markers including OCT4 (A), SOX2 (B), NANOG (C), SSEA4 (D) and TRA-1-60 (E). Scale bar: 100 μm. (F) Bisulphite sequencing analysis of the OCT4 and NANOG promoters in hiPSCs cocultured on hUC-MSC feeders and parental fibroblast line. White circles represented demethylated CpG dinucleotides; black circles represented methylated CpG dinucleotides. Cross represented sequencing was not achieved. The cell line is indicated to the left of each cluster.

Immunocytochemistry indicated the hiPSC colonies maintained on hUC-MSCs, still expressed stem cell markers OCT4, SOX2, NANOG, SSEA4 and TRA-1-60 even after 30 passages (Fig 5A–5E).

### Bisulphite genomic sequencing

Reprogramming of somatic cells is accompanied by demethylation of promoters of critical pluripotency genes. Therefore, bisulfite genomic sequencing was analysed to evaluate the methylation/demethylation statuses of cytosine guanine (CpG) dinucleotides in the promoter regions of pluripotent-associated genes, such as OCT4 and NANOG, revealed that they were highly demethylated, whereas the CpG dinucleotides of the regions were methylated in parental fibroblast line (Fig 5F). These findings indicate that these promoters are active in human iPS cells.

### *In vitro* and *in vivo* pluripotency of hiPSCs cocultured on hUC-MSCs

**Fig 6:**
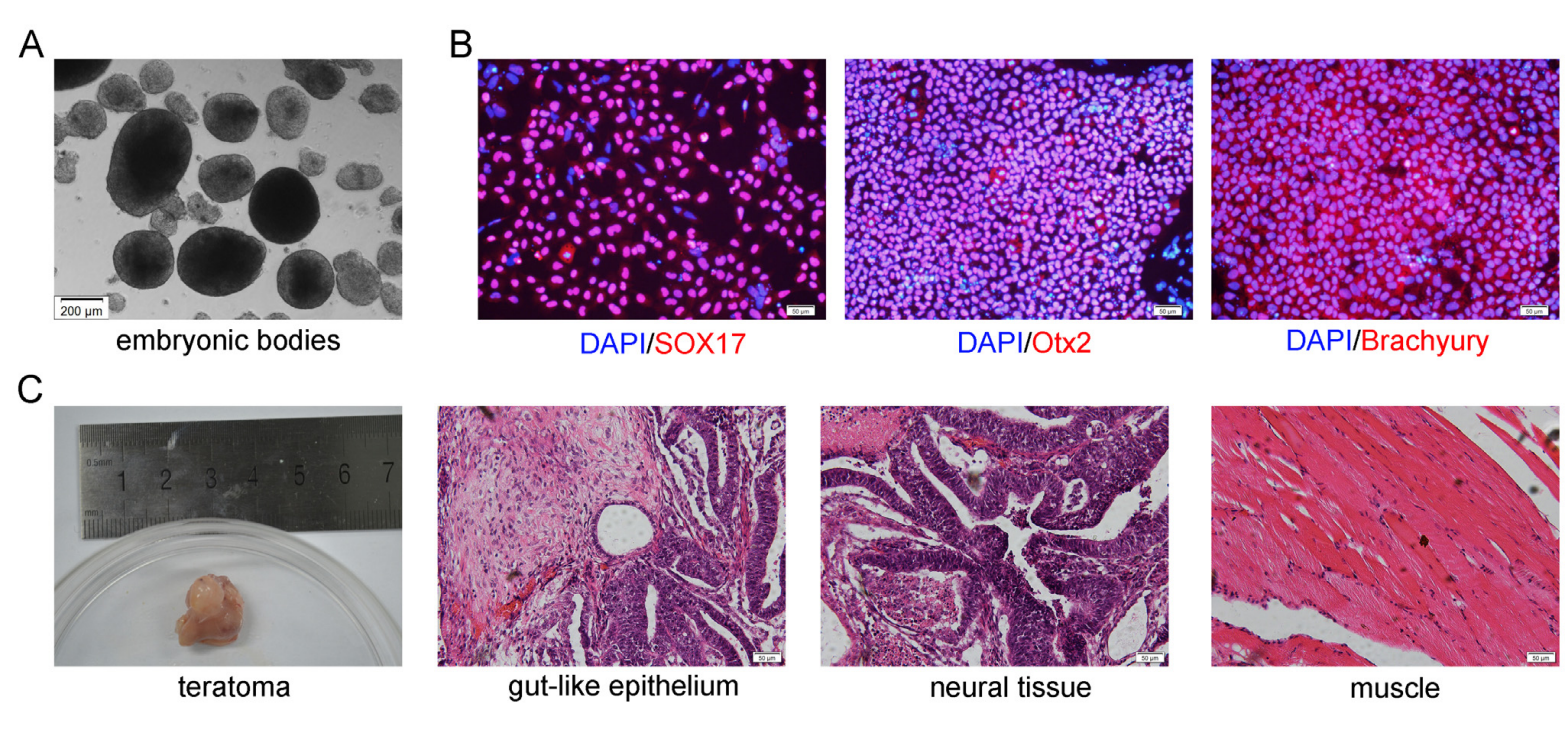
*In vitro* and *in vivo* differentiation of hiPSCs on hUC-MSCs feeder. (A) Morphology of EBs derived from hiPSCs cocultured hUC-MSCs feeder in floating culture at day 5. Scale bar: 200 μm. (B) Immunocytochemistry staining showed expression of three-germ-layer markers in hiPSC colonies on hUC-MSCs feeder *in vitro*: Endoderm (SOX17), ectoderm (Otx2) and mesoderm (Brachyury). Scale bar: 50 μm. (C) The HE staining showed the section of teratoma from hiPSCs on hUC-MSCs feeder are consisted of three germ layers: endoderm (gut-like epithelium), ectoderm (neural tissue) and mesoderm (muscle). Scale bar: 50 μm.

Like those maintained on MEF feeder, the hiPSCs on hUC-MSCs feeder were able to formed EBs when cultured in suspension (Fig 6A) and differentiate into derivatives of all three germline lineages when analyzed by endodermal (SOX17), ectodermal (Otx2) and mesodermal (Brachyury) marker expression in vitro under certain inductive conditions, demonstrating their pluripotent nature (Fig 6B).

The developmental potential of hiPSCs after long term culture on hUC-MSCs feeder was investigated in vivo using a xenograft model. In trials with different transplantations in NOD/SCID mice (N = 5), the formed teratomas consisting of derivatives of all three germ layers were observed in hiPSCs cocultured on hUC-MSCs (Fig 6C).

## Discussion

Feeder cells routinely used for hPSCs culture provide secreted factors, extracellular matix and cellular contacts, which are necessary for hPSCs to maintain their undifferentiated morphology and pluripotent status [30]. As the common feeder layers, MEF or SNL have been applied extensively to support self-renewal of hiPSCs and hESCs for in vitro cultures, but the traditional animal origin feeders unavoidably aggravate the risk of release of animal materials and unknown pathogens. Although feeder-free culture of hESCs has been reported, it may lead to chromosomal instabilities after long passages [31]. Large-scale production of hPSC derivatives goes hand in hand with the development of xeno-free environments excluding animal-derived products such as sera and feeders that are commonly used in traditional mammalian cell culture [32]. Considering the potential applications of hiPSCs needs a xeno-free culture system, we focused on the modification and improvement of the in vitro prolonged maintenance for hiPSCs.

hMSCs were shown to support ex vivo expansion of human hematopoietic stem/progenitor cells (HSPC) [33]. The ability was attributed to the production of various hematopoietic cytokines and stem cell factors [34], for example, hMSC cells secrete bFGF, while MEF cells do not. When cultured on MEF feeder cells, hESCs were found to express endogenous bFGF at the level around 0.1 ng/ml, and knockdown of the intracrine bFGF induced differentiation of hESCs [35]. Thus, hMSCs have recently been reported to support hESCs growth without exogenous bFGF [36, 37]. Such hMSCs were further proved to be the good adherent feeders from various tissues for maintaining hPSC colonies in their undifferentiated state and could be an alternative way to replace MEF feeder (Table 2) [23, 37–43]. Nonetheless, most tests were not authentic xeno-free feeder system because animal-derived serum was used in expansion and development of feeder cells. Ding et al. [23] had developed an animal-free feeder system from hUC-MSCs with human umbilical cord blood serum (CBS) instead of fetal bovine serum (FBS). However, regrettably the characteristics of hESCs continuously cocultured on hUC-MSCs were altered, such as gradually lossing the primary ability to differentiate into the three germ layers [23]. This hints that there are technical problems to develop xeno-free feeder system from hMSCs for the prolonged maintenance of hPSCs without any animal components (Table 2).

**Table 2:** Comparison of the coculture conditions with various origins of hMSCs feeder.

Article	Source of feeder	Culture condition	hPSCs	hPSCs passages	Teratoma formation
This report	human umbilical cord stroma	xeno-free medium	hiPSCs	over 30	Yes
Ma et al.[37]	human amniotic fluid	DMEM + 10% FBS	hESCs (X-01)	over 30	Yes
Fukusumi et al. [38]	human decidua	DMEM/F-12 + 10% FBS	hiPSCs	10–20	Yes
Soong et al. [39]	human amniotic fluid	IMDM + 15% FBS	hESCs	14–22	Yes
Havasi et al. [40]	human bone marrow	DMEM-low glucose + 10% FBS	hiPSCs	20	ND
Zhang et al. [41]	human bone marrow	DMEM-low glucose + 10% FBS	hiPSCs	14–16	Yes
Ding et al. [23]	human umbilical cord stroma	DMEM-low glucose + 10% CBS	hESCs (TW1)	over 20	No
Cho et al. [42]	human umbilical cord stroma	DMEM + 20% FBS	hESCs (H9)	20	Yes
Cheng et al. [43]	human bone marrow	DMEM-low glucose + 10% FBS	hESCs (H1)	under 13	ND

FBS: fetal bovine serum; CBS: human umbilical cord blood serum; ND: not determined because teratoma formation didn’t be mentioned in these reports.

The hUCs as the source of feeder cells have several advantages, such as wide availability, easy to handle and low immunogenicity [44, 45]. Umbilical cord is a good source of cells because hUC-MSCs are maintained in an early embryologic phase and therefore have retained some of the primitive properties of stemness [46]. MSCs that are derived from hUCs have been shown to be able to differentiate into neural-like cells, osteocytes, chondrocytes, adipocytes and others [18, 47–49]. Importantly, MSCs can suppress T cells independently of major histocompatibility complex (MHC) identity between donor and recipient because of their low expression of MHC-II and other co-stimulatory molecules [50]. The unique immuno-modulatory properties of MSCs have led to clinical trials of treatment of graft-versus-host disease (GVHD) after hematopoietic stem cell transplantation (HSCT) [51]. By means of this mechanism, the problems of immune rejection were not particularly concerned when using allogeneic MSCs, such as in hiPSCs cocultured with allogeneic MSCs feeder.

With this reprogramming system, we obtained ~3 hiPSC colonies from 1 × 10^4^ transduced HDF. This result is similar to the efficiency of the previously reported [1]. We have measured the reprogramming system with silence of the reprogramming factors and methylation status, these results indicated that the active and robust hiPSC colonies were generated from the reprogramming system. In this study, a xeno-free feeder-layer system was developed for prolonged expansion of hiPSCs in culture with hUC-MSCs feeder under xeno-free medium. These iPSCs carried on proliferation over 30 passages with undifferentiated morphologies when maintained on this xeno-free feeder. In this study, morphology, growth rate, chromosomology, cytochemistry and molecular biology analysis of hiPSCs cultured on hUC-MSCs feeder in comparison to those on MEF feeder confirmed the characteristics of hiPSCs were of pluripotent stem cell properties.

When using hUC-MSCs feeder instead of MEF feeder at initial stage, we found a number of hiPSC colonies were at first spontaneously differentiated within several passages. But remaining undifferentiated cells were again sustained cocultured with hUC-MSCs feeder. It implied that the microenvironment can selectively support the undifferentiated sections of hiPSCs in culture, which is provided by hUC-MSCs, indicating that hiPSCs kept an undifferentiated status on hUC-MSCs feeder as well as on MEF feeder in morphology once they adapted to the hUC-MSCs feeder. This adaption clearly did not compromise the pluripotency of hiPSCs, because hiPSCs that were cultured using hUC-MSCs feeder were strongly positive for markers that are also characteristic of undifferentiated hESCs (OCT4, SOX2, NANOG, SSEA4 and TRA-1-60), and could differentiate into all three germ layers in vitro and in vivo teratoma formation, even after a prolonged culture of more than 30 passages.

OCT4, SOX2 and NANOG are key transcription factors for regulating and maintaining of pluripotency of PSCs [52–54]. The levels of OCT4 are particularly critical for the state of hPSCs: loss of OCT4 results in the loss of pluripotency and differentiation into trophectoderm, while overexpression of OCT4 results in differentiation into primitive endoderm-like cells [55]. REX1 also plays an important role in pluripotency, proliferation and differentiation. REX1 can differentially affect various cellular processes, including translation, mitochondrial regulation, and cardiac differentiation [56]. In this study, the similar expression levels of OCT4, SOX2, NANOG and REX1 were found between hiPSCs cocultured with MEF feeder and hUC-MSCs feeder, implying that the pluripotency of hiPSCs was not primarily changed after prolonged cultured with hUC-MSCs feeder. Nevertheless, OCT4 was found to be down-regulated in hESCs cocultured with hUC-MSCs [23], this may be caused by the effect of culture conditions provided by hUC-MSCs, such as serum-dependence and secreted factors. MYC also maintains PSCs pluripotency and selfrenewal. ESC lines of MYC^knockout^ exhibited severely disrupted self-renewal, pluripotency, and survival along with enhanced differentiation [57]. Interestingly, the expression of MYC was more or less down-regulated on hUC-MSCs-maintained hiPSCs in this study. Previous study indicated maintained lower expression of stable MYC in ESC lines renders self-renewal and maintenance of pluripotency independent of LIF [58]. The maintenance of stemness and pluripotency of hiPSCs cocultured on hUC-MSCs was confirmed by qRT-PCR, in vitro differentiation and teratoma formation. Our results showed that over 30 passages they still maintained the similar properties and growth rate of undifferentiated hiPSCs cocultured on MEF, although trivial change in expression for undifferentiated gene could be observed (Fig 4B).

Recent report shows that the hMSCs could be used as feeder layers to support the differentiation of hiPSCs to hepatocyte-like cells [59], indicating that hMSCs not only maintained undifferentiated status of hPSCs, but also retained the potentials of differentiation. However it’s still not cleared that if hPSCs possess some particular differentiation tendency after continuously cocultured on MSCs feeder in comparison to those cultured on MEF feeder.

## Conclusions

Our present study demonstrated a feasible, potential, continuous and xeno-free feeder system for the sustained culture of undifferentiated hiPSCs by means of coculture with hUC-MSCs. As the stepping stone for stem cell research to further develop better and safer stem cells, the xeno-free culture system using hUC-MSCs as feeder layer for hiPSCs may be a good candidate for growth and expansion of hiPSCs for xeno-free culture. In next chapter, we will test whether hiPSCs can be reprogramed from adult somatic cells on hUC-MSCs feeder layer, and study if this feeder changes differentiation bias of hiPSCs after long time coculture.

## Supporting Information

S1 Fig A sample of original immunocytochemistry image included with this manuscript. (TIF)

S2 Fig A sample of original immunohistochemistry image included with this manuscript. (TIF)

S1 File The values used to build graph of Fig 1B. Each number represented the doubling time (hours) of different hUC-MSC lines.
(XLSX)

S2 File The values used to build graph of Fig 2A. Each number represented the number of spontaneously differentiated hiPSC colonies in observed 100 colonies.
(XLSX)

S3 File The values used to build graph of Fig 2B. Each number represented the number of spontaneously differentiated hiPSC colonies in observed 100 colonies cocultured on different hUC-MSC lines at passage X+1.
(XLSX)

S4 File The values used to build graph of Fig 2C. Each number represented the number of spontaneously differentiated hiPSC colonies in observed 100 colonies cocultured on different passages hUC-MSCs feeder at passage X+1.
(XLSX)

S5 File The values used to build graph of Fig 3C. Each number represented the doubling time (hours) of hiPSCs cocultured on different feeders at passage X+31.
(XLSX)

S6 File The values used to build graph of Fig 4A. Each number represented the relative expression of certain gene calculated with delta-delta Ct method. N/A: the Ct values of these groups can not be detected with this detection system owing to extremely low expression levels.
(XLSX)

S7 File The values used to build graph of Fig 4B. Each number represented the relative expression of certain gene calculated with delta-delta Ct method.
(XLSX)
