# Supplementary figures and images for: Development of a Xeno-Free Feeder-Layer System from Human Umbilical Cord Mesenchymal Stem Cells for Prolonged Expansion of Human Induced Pluripotent Stem Cells in Culture
Source: PLoS One. 2016 Feb 16;11(2):e0149023. doi: 10.1371/journal.pone.0149023 (PMC4755601; doi:10.1371/journal.pone.0149023)

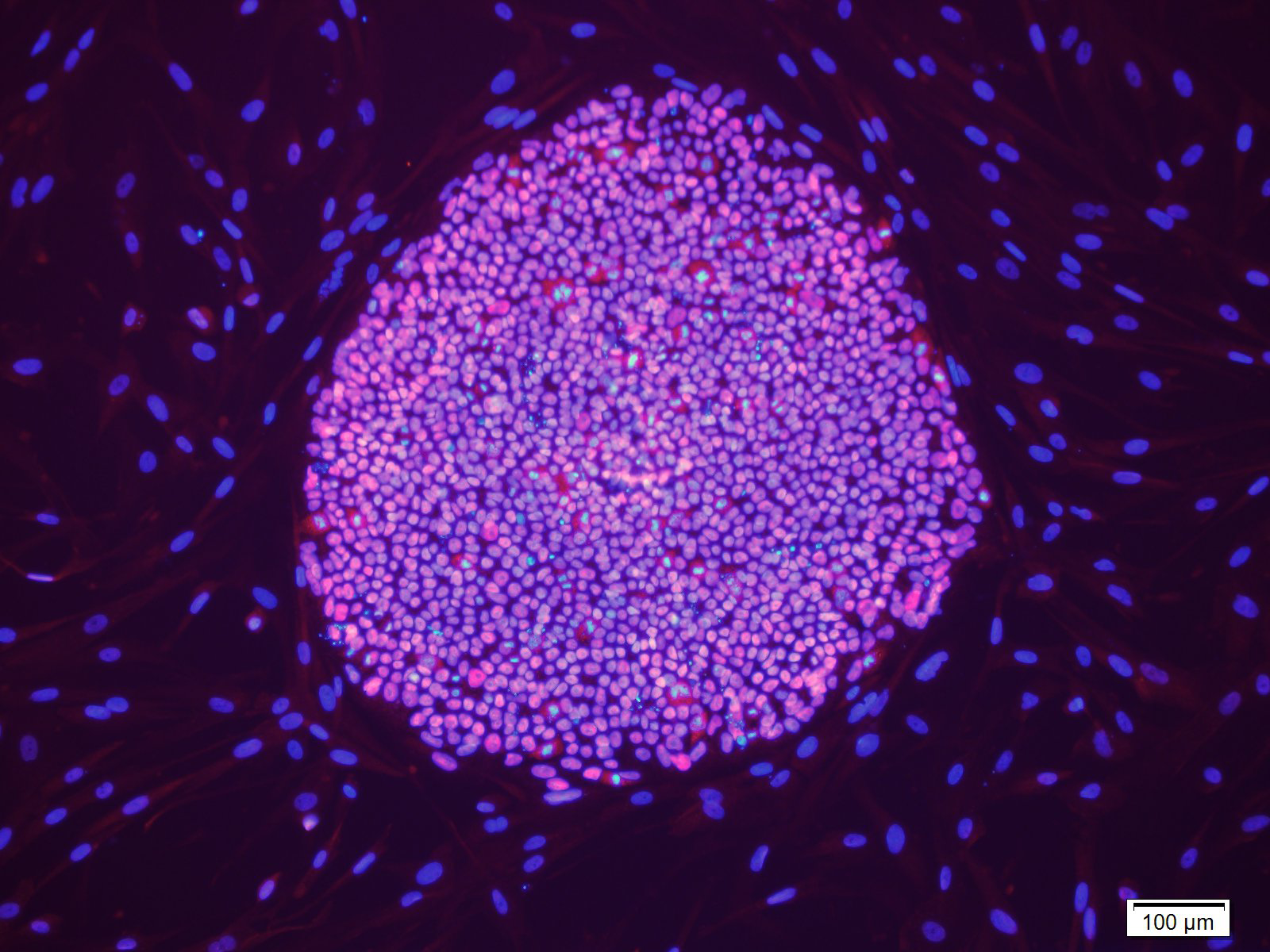

Supplement: S1 Fig — (TIF) [file pone.0149023.s001.tif]

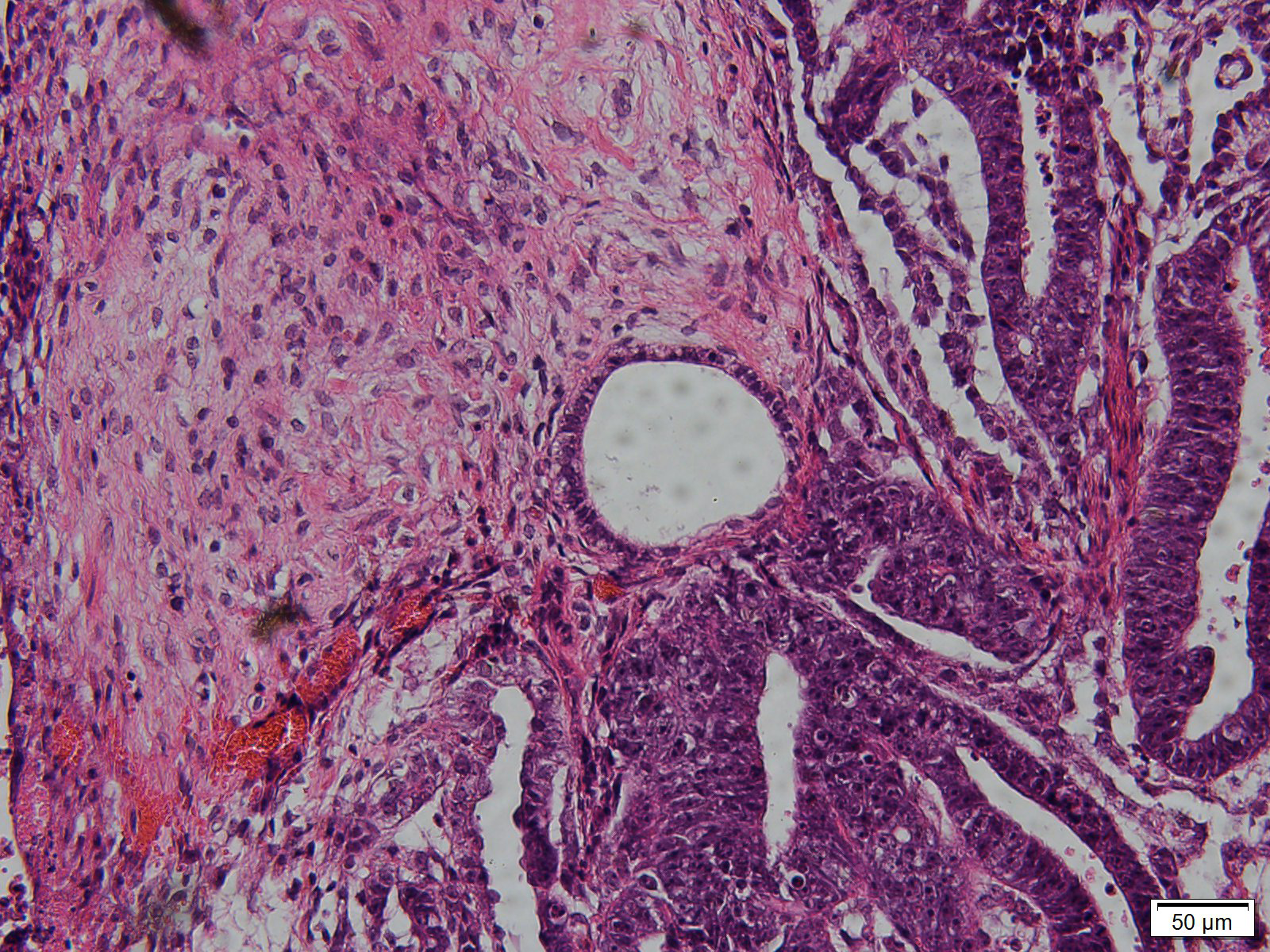

Supplement: S2 Fig — (TIF) [file pone.0149023.s002.tif]
